# Metal–Peptide Complexes as Promising Antibiotics to Fight Emerging Drug Resistance: New Perspectives in Tuberculosis

**DOI:** 10.3390/antibiotics9060337

**Published:** 2020-06-18

**Authors:** Concetta Di Natale, Ilaria De Benedictis, Arianna De Benedictis, Daniela Marasco

**Affiliations:** 1 Department of Pharmacy, University of Naples “Federico II”, 80134 Napoli NA, Italy; concetta.dinatale@unina.it (C.D.N.); ilariadebenedictis2308@gmail.com (I.D.B.); ariannadebenedictis@gmail.com (A.D.B.); 2 Center for Advanced Biomaterial for Health Care (CABHC), Istituto Italiano di Tecnologia, 80125 Naples, Italy; 3 Interdisciplinary Research Centre on Biomaterials (CRIB) and Dipartimento di Ingegneria Chimica, dei Materiali e della Produzione Industriale, University of Naples Federico II, Piazzale Tecchio 80, 80125 Naples, Italy

**Keywords:** metallopeptides, antimicrobial peptides, *Mycobacterium tuberculosis*, drug delivery

## Abstract

In metal-peptide interactions, cations form stable complexes through bonds with coordinating groups as side chains of amino acids. These compounds, among other things, exert a wide variety of antimicrobial activities through structural changes of peptides upon metal binding and redox chemistry. They exhibit different mechanisms of action (MOA), including the modification of DNA/RNA, protein and cell wall synthesis, permeabilization and modulation of gradients of cellular membranes. Nowadays, the large increase in antibiotic resistance represents a crucial problem to limit progression at the pandemic level of the diseases that seemed nearly eradicated, such as tuberculosis (Tb). *Mycobacterium tuberculosis* (*Mtb*) is intrinsically resistant to many antibiotics due to chromosomal mutations which can lead to the onset of novel strains. Consequently, the maximum pharmaceutical effort should be focused on the development of new therapeutic agents and antimicrobial peptides can represent a valuable option as a copious source of potential bioactive compounds. The introduction of a metal center can improve chemical diversity and hence specificity and bioavailability while, in turn, the coordination to peptides of metal complexes can protect them and enhance their poor water solubility and air stability: the optimization of these parameters is strictly required for drug prioritization and to obtain potent inhibitors of *Mtb* infections with novel MOAs. Here, we present a panoramic review of the most recent findings in the field of metal complex-peptide conjugates and their delivery systems with the potential pharmaceutical application as novel antibiotics in *Mtb* infections.

## 1. Introduction

Tuberculosis (Tb) is an airborne infectious disease caused by the bacillus *Mycobacterium Tuberculosis* (*Mtb*). Unfortunately, even though several therapies have been developed, tuberculosis still has a high incidence and mortality rate. Current drugs and vaccines have no significant impact on its control; indeed, Tb incidence rates are now higher than 25 years ago. According to the World Health Organization (WHO), every year, 10 million people get sick. Despite being a preventable and curable disease, 1.5 million/year die of *Mtb* infections, making it the world’s top infectious killer [1]. The emergence of drug-resistant Tb (multidrug-resistant (MDR-Tb) and extensively drug-resistant (XDR-Tb)) is considered a public health crisis, with several strains actually resistant to all available drugs [2]. The *Mtb* cell wall has a peculiar conformation that concurs to the onset of MDR, since it is characterized by features belonging both to Gram-positive and -negative cell envelopes: it has a peptidoglycan layer almost as thick as in Gram-positive bacteria and an outer layer composed by glycolipids similar to the external membrane of Gram-negative bacteria [3]. In detail, the mycobacterial wall is called a myco-membrane and presents an uncommon thickness, even though constituted of different layers, each with a different hydrophobicity, in dependence of the diverse composition of lipids and sugars. Indeed, moving from the external surface toward the inner cytoplasmic membrane, different structural polymers are observable: glycolipids, mycolic acids, arabinogalactans, peptidoglycans, and lipomannans [4]. Peptidoglycan and arabinogalactan are hydrophilic layers that prevent the passage of hydrophobic drugs and are covalently linked to an external layer of mycolic acids and wax-D (macromolecular peptidoglycolipids) moieties that in turn, being mainly hydrophobic, form a barrier that encumbers the penetration of hydrophilic molecules into bacteria [5]. Thus, the permeability of the wall is highly restricted and the antibiotics have difficulty passing through this barrier, and many antimicrobial compounds are active against mycobacteria only at high concentrations [6].

A promising field to answer to drug resistance in the pharmaceutical research is the development of novel and peptide-based compounds as therapeutic antibiotics. Often, peptides demonstrated elected compounds to target proper and difficult targets as protein complexes for their high specificity as well as recent and innovative synthetic strategies [7]. Several natural antimicrobial peptides (AMPs) have been identified over time and many improvements of their biological activity were achieved through their ad hoc chemical modifications. Among these variations, the coordination of the transition metal can be included: the presence of a metal ion can alter the AMP’s structure, but also provide redox action on targets; this strategy is also used to interfere with the metals belonging to bacteria. A crucial point in overcoming drug resistance is the full comprehension of the way of action of known drugs and how it can be improved by the presence of metal cofactors for the design of novel metal antibiotics based on natural AMPs. This review aims to discuss modified AMP structures and their MOAs, focusing on the potentialities of their therapeutic use in Tb infection.

### Antimicrobial Peptides

AMPs are usually short peptide sequences endowed with a broad range of activity against both Gram-positive and -negative bacteria; many of them also show anti-biofilm activity [1,8] through inhibition of biofilm formation and adhesion or disruption of pre-formed biofilms and down-regulation of quorum-sensing factors. AMPs can exhibit remarkable levels of specificity and genetic variability at the level of single amino acids, can dramatically alter resistance to infection. The pharmacodynamics and MOAs of AMPs are much more favorable than those of conventional antibiotics for the prevention of resistance evolution [9].

Natural AMPs belong to the innate immune system in humans, animals and plants [10,11], they combat infections by influencing the host’s immune responses and limiting enhanced inflammation [12]: they can lead to bacterial cell death through both membranolytic and non-membranolytic mechanisms or the interaction with intracellular targets, such as DNA, RNA, and proteins. AMPs are characterized by both hydrophobic and hydrophilic domains and are typically short (less than 50 amino acid residues) and strong cationic character (for this, they are often called cationic antimicrobial peptides (CAMPs)) [12,13]: hence, the positive net charge ensures better interaction with negatively charged bacterial membranes. CAMPs can differ significantly in sequence and structure and are classified on the basis of their conformations [12]; usually, they accumulate at the cellular surface and, once a certain concentration is reached, they assemble on the bacterial membrane. In the barrel-stave model of the membrane penetration mechanism, peptides perpendicularly enter the membrane, promoting lateral peptide-peptide interactions. In this mechanism, the amphipathic structure of AMPs is crucial, since hydrophilic residues form the channels’ lumen, while the hydrophobic side establishes favorable interactions with lipoid membranes [14].

The main classes of CAMPs in vertebrates are defensins and cathelicidins. Defensins have a common β-sheet core stabilized with three disulfide bonds between six conserved cysteines and are subdivided into α-, β-, and θ-defensins on the basis of the linkages of Cys [12,15,16], while most cathelicidins are α-helical, amphipathic, with a hydrophobic surface that favors the interaction and perturbation of membranes with anionic surfaces [12]. Other cathelicidins comprise β-hairpin peptides (12–18 residues) and linear sequences (13–39 residues), that are involved into immunomodulation of the inflammatory response [17,18,19].

Several natural AMPs also demonstrated their antimicrobial activities against *Mtb* and, more recently, on the basis of investigated MOAs, many synthetic peptides have been designed and tested against various mycobacteria strains, providing improved and novel antimicrobial activities [20]. Several examples of both natural and chemically modified AMPs are reported in Table 1 [21]. Among the investigated models, the anti-mycobacterial compound produced by *Pantoea dispersa* W18 named pantocin wh-1 is a short peptide of 16 residues endowed with a cyclic structure, which demonstrated anti-Tb activity both in vitro and in vivo [22]. The screening of a library of small sequences (~10 amino acids) designed using a collection of 253 natural CAMPs active against *Pseudomonas aeruginosa* and optimized in silico provided several promising compounds with low toxic effects [23] against *Mtb*. Five of the most active sequences were modified by inserting cinnamic acid to the N-terminal side and then tested in vitro: they proved more effective against *Mtb* strains than unmodified parental peptides [24].

Two long synthetic peptides (~30 residues) designed using the β-defensin structure inhibited growth of the H37Rv and MDR *Mtb* strains, but they do not provide hemolytic effects on blood cells [25]. Furthermore, two plectasin derivatives named NZ2114 and NZX demonstrated anti-microbial activity in comparison with parental LL-37, a well-known cathelicidin sequence: NZX was the most effective, since it exhibited an inhibition grade of the mycobacterial growth of 74%, effectively killed *Mtb* in vitro, and decreased the bacterial load in vivo [26]. Two AMPs derived from the venom glands of the Mexican scorpion *Vaejovis punctatus* named VpAmp1.0 and VpAmp2.0 (19, 25 residues long) were reduced in size in several smaller synthetic peptides that effectively inhibited the growth of two clinically isolated strains of *Mtb*, including the MDR one, with low minimum inhibiting concentrations (MICs) [27]. Noticeably, several lucky studies outlined a clear MOA as in the case of optimized derivatives of griselimycin that proved highly active against *Mtb* both in vitro and in vivo by inhibiting the DNA polymerase sliding clamp dnaN [28]; conversely, lassomycin binds to an acidic region of the ATP-dependent Clp protease (ClpC1) and markedly stimulates its ATPase activity without stimulating catalyzed proteolysis (ClpP1P2), which is essential for the viability of *Mtb* [29].

Synergistic effects against *Mtb* strains between AMPs and conventional tuberculosis drugs were observed in several studies. Bacteriocin AS-48 produced by *Enterococcus faecalis* demonstrated interesting antimycobacterial activity alone that is enhanced if used in combination with a lysozyme or ethambutol [30]. Similarly, two synthetic 11-residue peptides derived from the *Naja atra* cathelicidin (ATRA-1A and ATRA-2) that bear variations of a repeated motif and one derived from the human β-defensin hBD3 (hBD3-Pep4) demonstrated synergistic effects with rifampicin (Rf), above all against *Mycobacterium smegmatis* [31].

More recently, a new therapeutic approach has emerged to treat tuberculosis, host-directed therapy (HDT), which aims to induce immune modulation. It involves agents that are not microbicides per se, but can additively or synergistically enhance activity of the anti-Tb drugs. HDT can be used to improve long-term functional outcomes of respiratory tract infections (RTIs), such as MDR-Tb [32]. For example, in a Tb infection, *Mtb* down-regulates the expression of LL-37 in human macrophages; this reduction can be counteracted by treating the infected macrophages with vitD3, phenylbutyrate (PBA), or their combination [33]. Indeed, 1,25(OH)_2_-vitamin D modulates cathelicidin and defensin gene expression in innate immune cells, such as monocytes and macrophages [34].

## 2. Metal-Based Antibiotic Therapeutic Agents

### 2.1. Antimicrobial Metal Complexes

Metal-based compounds represent promising potential new drugs for different diseases: cancer, neurodegeneration, and inflammation [52,53,54,55].

In the antimicrobial field, metal complexes associated with known AMPs often present different MOAs with respect to single peptides: the destruction of bacterial plasma membranes as well as hydrolytic or oxidative cleavage of nucleic acids promoted by metal-based compounds has garnered significant interest as a result of their broad range of application [56].

**Figure 1:**
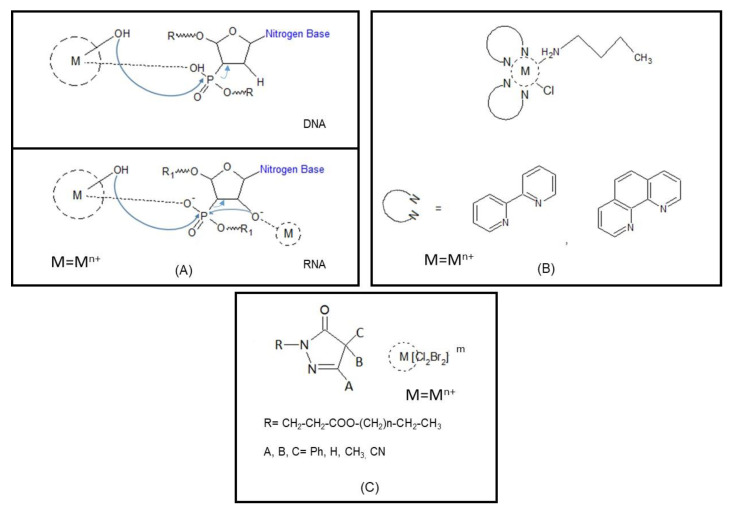
Schematic representation of DNA/RNA cleavage catalyzed by metal complexes; arrows indicate the nucleophilic attack and leaving groups during substitution, M^n+^ = mostly divalent cations (**A**) assisted by metallosurfactants with bidentate N,N ligands (**B**), and (**C**) with different substituents, M^n+^ = mostly in octahedral and tetrahedral coordination geometry, m = the net charge of the complex, including surfactant contribution.

Nuclease function is involved in a variety of biological functions, including nucleic acid synthesis, recombination, regulation, processing, and degradation [57,58,59,60], hence the development of low molecular weight metal complexes as “artificial nucleases” is of crucial importance [61]. For most natural metallonucleases, Mg^2+^ is the best candidate ion due to its high natural availability and Lewis acidity, but several examples involving Zn^2+^, Ca^2+^, and other metals have been reported [62,63]: in these studies, the metal ion promotes hydrolysis of the nucleic acid phosphate backbone [64]. The rate of cleavage depends on the metal and on the formation of the metal-bound hydroxide nucleophilic intermediates. An alternative approach in the degradation of nucleic acids consists of an oxidative cleavage in the presence of redox-active ions through intermediates that produce reactive oxygen species (ROS) followed by electron transfer (ET) from the metal to molecular oxygen or peroxide. The oxidized metal is then reduced by ascorbate or dithiothreitol (DTT) to continue ROS formation, which typically abstracts hydrogen from the deoxyribose/ribose ring followed by spontaneous cleavage of C–C and C–O bonds (Figure 1A) [65]. Efficient synergy derives from DNA cleavage coupled to the inhibition of biofilm formation [66]. Recently, the ability of passivated gold nanoparticles (NPs) with multiple Ce^4+^ complexes on the surface of colloidal magnetic particles to degrade extracellular DNA (eDNA) and inhibit biofilm formation was reported [67].

Metal complexes with amphiphilic character called metallosurfactants exhibit precise properties: they present long alkyl chains (hydrophobic part) and metal ions coordinate, mostly, ligands with nitrogen donors (hydrophilic fragment). Due to the presence of different functional regions, they can be soluble both in water and non-polar solvents and can be adsorbed both at fat/water or air/water interfaces; and, as other surfactants, they can self-assemble to form aggregates after reaching the critical aggregation concentration (cac) [68]. Interestingly, metal complexes with amphiphilic molecules demonstrated ability to penetrate cells more easily than the ligands alone [69]: in these, donor atoms present high electron density increasing the hydrophobicity of the system, thus the adsorption on the lipidic cell walls occurs with less difficulty, allowing easy passage through the bacterial membrane. An excellent example is related to antimicrobial Co^3+^ complexes bearing as ligands N,N-donor ligands, such as ethylenediamine (en), bipyridine (bipy), phenanthroline (phen), or triethylenetetramine (trien), with a long chain amine ligand (Figure 1B). These metallosurfactants demonstrated ability to bind DNA and to inhibit growth of some bacterial and fungal species [70].

Similarly, square planar complexes of different ions including long alkyl chains with amine functionalities aiding the formation of bilayers or metallomicelles demonstrated activity against various pathogenic microorganisms: hemolysis assay demonstrated different toxicities (Fe > Ni > Co) at concentrations below the critical micelle concentration (cmc) [71]. Pyrazolinone and pyrazolone moieties as surfactants were coordinated through an ester bond with long alkyl chains and Sn and Cu were used as counter ions (Figure 1C): while the cationic surfactants themselves already showed moderate antibacterial activity as salts, an important increase in activity was achieved (Sn > Cu). The authors correlate the activity of the metal complexes with their electronegativity with respect to a single molecule [72]. Also, benzaldehyde derivatives condensed with amines with long alkyl chains demonstrated a modest activity against Gram-positive and -negative bacteria strains as well as fungi; this activity resulted in the mobility increased by complexation of transition metal ions, in particular, of Co, Cu, and Mn. Similarly, in these complexes, the high electron density within the ligand increased the hydrophobicity of the molecules if coordinated to metal ions [73].

### 2.2. Metal-Antimicrobial Peptides

Among metal complexations of AMPs, an intensely explored motif is Amino Terminal Cu(II)- and Ni(II)(ATCUN), a Cu^2+^- and Ni^2+^-binding sequence with an XXH stretch usually as an amino terminal, where X is any amino acid [74]. Piscidins are a family of AMPs found in the teleosts; two of them, P1 (**FFH**HIFRGIVHVGKTIHRLVTG) and P3 (**FIH**HIFRGIVHAGRSIGRFLTG), are linear peptides that exhibit broad antibacterial activity, although their MOAs are quite different [75,76]: in detail, P3 proved less toxic to mammalian cells and more active on biofilms and persister cells. The ATCUN motif is Phe-Phe-His- for P1 and Phe-Ile-His- for P3 and binds Cu^2+^ and Ni^2+^ ions with high affinity: the α-amine group, the two amide nitrogens of the backbone, and the δ-nitrogen of the third His form the ligand field with a planar coordination geometry [77]. Both P1 and P3 cross bacterial membranes and co-localize with intracellular DNA, but P3 is more condensing to DNA, while P1 is more membrane-active, and the bactericidal effects of P3 are enhanced in the presence of Cu^2+^ caused by the formation of radicals (superoxide O_2_, hydroxyl radical HO) that nick DNA [78]. Interestingly, progressive loss of the plasmid supercoiled form pUC19 and an increase of the nicked and linearized forms of DNA were outlined during the time-dependent cleavage of the plasmid incubated with P3 in the presence of H_2_O_2_ and ascorbic acid [76].

More recently, in a study related to the *Clostridioides difficile* infection, a hospital-acquired disease highly resistant to multiple classes of antibiotics, it has been pointed out that P1, P3 activities strongly depend on the anaerobic conditions corroborating the hypothesis related to the formation of radical species during their MOA [79]. Ovispirin 3 (OV-3) is a synthetic AMP derived from the sheep myeloid antimicrobial peptide (SMAP-29) [80], a cathelicidin member, with antimicrobial activity against a variety of pathogens [81,82]. Metallopeptide analogs of OV-3 exhibited DNA/RNA binding and nucleasic activity in vitro and higher antimicrobial activity with lower MIC values towards *Escherichia coli* [83].

The ATCUN motif also naturally occurs in histatin-5 (Hst-5) [84], a 24-residue sequence found in saliva that binds Cu^2+^ with a dissociation constant *K_D_* in the picomolar range [85], or hepcidin (Hpc-25), a 25-residue peptide involved in iron homeostasis for which *K_D_* has a micromolar value [86]. Hst-5 is a conformationally dynamic peptide, with bioactive forms preferentially stabilized by zinc interactions that make it responsive to the coordination of other divalent metal ions, with distinct copper and zinc sites [87]. At the same time, Hpc-25 was identified as the main iron regulator in the human body through the binding to the iron exporter ferroportin: the N-terminus of Hpc is responsible for this interaction and with the same motif, in turn, it is able to bind Cu and Ni enhancing its antimicrobial power [88]. Other examples of an ATCUN sequence are Asp–Ala–His in serum albumin (HSA) and Gly–His–Lys present in several wound-healing factors: encouraging reported results open the way to add a high-affinity Cu-binding site to almost any peptide or protein by chemical or recombinant approaches [89]. Noticeably, XXH motifs have already been grafted to short AMPs (such as anoplin or buforin), which act by interacting with the cell membrane [90] or inhibiting intracellular targets, revealing a powerful strategy to increase their biological activity [91].

Sub5 is a short synthetic AMP derived from the screening of substitution library of bactenecin 2 [92] and the addition of an ATCUN motif to Sub5 showed a 2- to 3-fold increase in antimicrobial activity for a variety of microbes, with MICs and K_D_ values of DNA7RNa binding in the low micromolar range [93]. On the other hand, several other systems intrinsically need the presence of metal ions: mucin 7 (named MUC7 or MG2) is a salivary protein whose fragments exhibited strong antimicrobial activities acting as a natural multicomponent protector of organisms against pathogens; its MOA implies the presence of metals. Recently, several shorter versions of Sub5, 12-mer peptides containing both or alternatively His^3^ and His^8^ for the coordination to metals were investigated against bacterial and fungal strains revealing activity against *Enterococcus faecalis* and *Staphylococcus epidermidis* enhanced upon Cu^2+^ or Zn^2+^ addition. Coordination studies revealed the formation of mainly mononuclear complexes in a solution in which the binding affinity appeared inversely proportional to the antimicrobial properties indicating a tunable balance between K_D_ and MIC values [94].

Moreover, biophysical studies strongly aid the definition of MOAs, for example, bacitracin A, a cyclic peptide produced by *Bacillus subtilis* and *licheniformis*, exhibited the ability to bind to diverse divalent metals with potential different effects [95]: the Mn–bacitracin complex showed enhanced inhibition of microbial growth of Gram-positive *Staphylococcus aureus* and *Enterococcus* spp., but also a potent SOD-like (superoxide dismutase) activity suggesting the ability to continue functioning under stress. The crystal structure of the Zn^2+^ complex exhibited an octahedral coordination geometry in which ligands are constituted by the N-terminus of the peptide, Glu^4^, and thiazoline nitrogen [96]. Dermicidin is secreted from eccrine sweat glands and proteolytically processed into two fragments, dermicidin-1 and 1-L (DCD-1 and DCD-1L), active against *E. coli*, *Enterococcus faecalis*, and *S. aureus*. Circular Dichroism (CD) studies revealed that peptide fragments adopt α-helical conformations both in sodium dodecyl sulfate (SDS) [97] and TFE (2,2,2-trifluoroethanol) and oligomerization was observed for DCD-1L in a process stabilized by the Zn^2+^ ion, suggesting that it could also occur in vivo [98,99].

Furthermore, clavanin A (VFQFLGKIIHHVGNFVHGFSHVF), a 23-residue AMP that shows an α-helical conformation in a hydrophobic environment, exhibited a wide range of antimicrobial functions that are mainly exerted through liposome permeabilization and appeared enhanced upon the coordination of Zn^2+^ to His^17^ and His^21^ [100].

On the other hand, transition metals appear essential nutrients for pathogens and mammals through “nutritional immunity” that limits their availability [101]. Furthermore, hosts are also able to secrete antimicrobial peptides, such as microplusin, psoriasin, and calprotectin, that inhibit bacterial infection through metal binding.

Microplusin structurally presents three disulfide bonds and five α-helices and is active against Gram-positive bacteria, but not Gram-negative ones. This can be justified by different physiological levels of copper; indeed, the addition of copper changes its effects toward *Micrococcus luteus* and *Cryptococcus neoformans*, suggesting that it functions by sequestering copper from pathogens [102]. Calprotectin is a member of the S100 family that undergoes heterodimerization through the coordination of metal ions, above all, of zinc and manganese, but more recently, it has been demonstrated that calprotectin’s activity and metal binding can be further influenced by the presence of calcium, which improves the affinity for both zinc and manganese [103].

An unusual, but essential for *Mtb* cytochrome P450 metallo-enzyme (CYP) named CYP121A1 catalyzes the oxidative coupling of two tyrosine residues in the substrate cyclodityrosine (cYY) to generate a C−C bond: this process seems to be strictly related to the biosynthesis of glycopeptide antibiotics, such as vancomycin and teicoplanin. CYP121 is not found in other microorganisms and appears a valid target for the development of specific agents against *Mtb* [104].

### 2.3. Antibiotic Metal Complexes in Mtb Treatment

**Figure 2:**
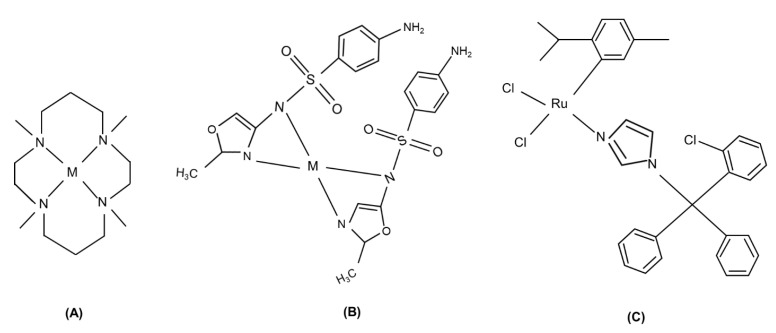
Several examples of metal-drug complexes: (**A**) cyclam derivatives, M = M^n+^, (**B**) sulfonamide complexes, M = M^n+^, (**C**) ruthenium-clotrimazole metal complex.

Over the last years, the metal coordination of the antibiotics already known has been pursued to overcome problems connected to impeding ways: the recent studies with *Mtb* demonstrated that metal complexes of isoniazid act preferentially against drug-resistant strains [105]. Similarly, a class of functionalized 1,8-disubstituted cyclam metal derivatives displays (i) low micromolar activity against MDR-*Mtb*, (ii) no toxicity to human cells, and (iii) inhibition of intracellular growth [106]. Derivatives of 1,8-naphthalimide bearing two pendent groups as ligands produced a series of metal complexes endowed with micromolar antimycobacterial activity: most lead compounds contain a metal ion (Cu^2+^ or Zn^2+^) coordinated to the macrocycle [106] (Figure 2A).

Sulfadoxine is a sulfonamide used in combination with pyrimethamine to treat malaria caused by the *Plasmodium falciparum* parasite [107]. Pyrimethamine and sulfadoxine target two enzymes that are crucial to the parasitic folate biosynthetic pathway, i.e., dihydrofolate reductase (DHFR) and dihydropteroatesynthetase (DHPS). With respect to this, cobalt, copper, nickel, and zinc complexes of the sulfonamide 4-[(E)-(5-bromo-2-hydroxyphenyl)methylidene]-amino-N-(4,6-dimethyl-pyrimidinyl)benzenesulfonamide revealed moderate to significant antibacterial activity against one or more bacterial strains and good antifungal activity [108] (Figure 2B).

Several ruthenium complexes containing the 2-pyridinecarboxylic acid anion (picolinate) already demonstrated potential anti-Tb activity [109,110]; more recently, novel arene Ru, cyclopentadienyl (Cpx) Rh and Ir complexes containing an N,N’-chelated pyridylimino or quinolylimino ligand functionalized with the sulfadoxine were tested: in these complexes, the imino and pyridyl/quinolyl nitrogens provided an N,N-chelation site for the metal and exhibited potent antiplasmodial activity with IC_50_ values in the low micromolar range. An increase in the size of both the Cpx ligand and the aromatic imino substituent increased hydrophobicity and antiplasmodial activity [111].

Sulfonamide copper complexes presented antibacterial activity; their general formula is as follows: [Cu(sulfa^−^)_2_(N^^^N)], where sulfa is the anionic form of a sulfonamide ligand (sulfameter (mtrH) or sulfadimethoxine (sdmx)) and N^^^N is bipy or phen; they exhibit different geometry and oligomeric state depending on ligand hindrance and demonstrated the ability to bind DNA [112] and good anti-*Mtb* activity in the micromolar range [113].

Clotrimazole (CTZ) an old antifungal agent with high efficacy and minimal side effects [114], showed antitumoral effects and lower cytotoxicity in complex with ruthenium (RuCl_2_(CTZ)_2_)in comparison with platinum complexes [115]. Recently, different Ru/phosphine/diimine complexes demonstrated a promising activity against *Mtb*; in particular, three new complexes with the general formula [RuCl(CTZ)(bipy)(P-P)]PF_6_, where P-P can be 1,2-bis(diphenylphos-phino)ethane, 1,4-bis(diphenylphosphino)butane, and 1,1′-bis(diphenylphosphino)ferrocene, were analyzed. These complexes generated hydrophobic interactions with proteins and DNA causing anti-*Mtb* activity with the MIC values equivalent or enhanced if compared to free CTZ, cycloserine, gentamicin, tobramycin, and clarithromycin [116], (Figure 2C).

Similar good results were obtained by diphosphine-derivative Ru-complexes; bis(diphenylphosphino)amines are donor ligands containing a single nitrogen atom between two phosphorus atoms (P-N-P) able to form a quaternary chelating ring for a metal center with the following general formula: [RuCl(η^6^-p-cymene)(P-N^R^-P)]X, where R can be = CH_2_Py, CH_2_Ph, Ph, p-tol, and X = PF_6_^−^ or BF_4_^−^. In the antimicrobial mechanism against *Mtb*, a crucial role appeared exerted by the basicity of the amines, which can affect the donor ability of the P-N-P ligands [117].

Ternary copper complexes with the general formula Cu(N-O)(N-N)(ClO_4_)_2_, in which N-O = 4-fluorophenoxyacetic acid hydrazide (4-FH) or 4-nitrobenzoic hydrazide (4-NH) and N-N = phen, 4–4′-dimethoxy-2-2′-bipyridine (dmb) or bipy demonstrated interesting antimicrobial activity: they displayed an octahedral distorted geometry around the copper ion in which both ligands are coordinated in a bidentate mode, (N-O) and (N-N). CD analysis revealed their ability to bind DNA [118] to explain their MOAs and, in comparison with similar compounds containing aromatic diimine ligands (N-N), they appeared the most active [118,119,120].

An interesting antimicrobial compound endowed with potential is the 8-hydroxyquinoline (8HQ): recently, a high-throughput screening identified ~200 8HQ derivatives exhibiting micromolar MIC values against *Mtb*; this activity appeared to be enhanced by its ability to chelate metal ions as Cu^2+^ in a 1:1 complex [121].

More in general, copper is able to enhance the antimicrobial activity of other basic antibiotics, such as viomycin and capreomycin, which are the most effective agents against MDR-Tb. The Cu–viomycin complex is stabilized by H-bonds able to induce helical conformation into the peptide and is able to cause strong DNA degradation [122]; this mechanism is associated with the modulation of the antigenomic delta ribozyme catalytic activity [123].

Similarly, copper is able to enhance the antimicrobial activity of isoniazids (INH) that represent the most commonly used drugs in Tb [124]: in the study of related MOAs, they exhibited the ability to form lipophilic vehicles for the delivery of the undamaged INH moiety into bacteria [125].

Four INH–copper complexes with different INH derivatives, such as 2-pyridinecarboxaldehydeisonicotinoyl hydrazone (HPCIH) 2-acetylpyridine-(HAPIH), 2-pyridineformamide-(HPAmIH), and pyrazineformamide-(HPzAmIH), exhibited different and controversial performance towards *Mtb* [125]; the same compounds coordinated to Ag^+^ did not exhibit substantial enhancement of the anti-*Mtb* activity [126].

Very recent promising results have been obtained by a novel class of Cu and Co compounds containing benzohydroxamate as a ligand: the investigated compounds interact with the enzyme urease, which is crucial for the bacillus survival in the intraphagosomal environment [127].

Furthermore, Pd complexes with capreomycin (C), kanamycin (K), and ofloxacin (Ofx) have been characterized: NMR studies revealed that in the C–Pd compound, different species are present and the tetra hydropyrimidinic is the prevalent one: for K–Pd, proton nuclear magnetic resonance (^1^HNMR) spectra present an overall broadness indicating that two Pd ions can contemporarily or singularly bind two different sites, similar to the Ofx–Pd complex [128]. Ferrocene derivatives appear to have a great impact as antiproliferative compounds in breast cancer and infections [129]: they generate ROS due to the presence of the redox-active Fe^2+^ [130]: recently, nine different isonicotinyl and pyrazinyl ferrocenyl-based complexes have been characterized and analyzed and only mononuclear complexes exhibited antimicrobial action against Tb [131].

## 3. Delivery Systems for Antibacterial Compounds in Tb

Various drug delivery systems have been exploited over the last years in the therapeutic strategies related to Tb: metal NPs of Ni, Ag, Au [132,133], metal complexes of antibiotics [134], as well as polymers with antimicrobial properties, both decorated with AMPs and not. This latter strategy has the advantage to combine synergistically the effects of bioactive polymer matrices with drugs [135,136,137].

Most of used delivery nanosystems summarized in Table 2 include liposomes, metal/polymeric/multifunctional NPs, dissolvable hydrogel microneedles [138,139]. For example, liposomes composed of both lipidic and aqueous fractions are used for the delivery of amphiphilic molecules, while multifunctional NPs (based on silver, titanium, gold) are applied to entrap, couple, or adsorb AMPs combining their antimicrobial activity with that of the peptides [140].

The LL-37 peptide, once encapsulated in lipid nanostructures [141] or coated onto magnetic Ni NPs, maintained its antimicrobial activities with longer times of action [142]; Au and Ag [143] NPs conjugated with both thioalkenes and lipo-surfactin or lactoferrin exhibited enhanced antimicrobial activities with respect to peptides alone [98,144]; in these studies, NPs seemed to aid the contact of AMPs with bacterial membranes [98,145] causing DNA damage through the production of ROS [146].

Ru/phosphine/diimine complexes, lichexanthone derivates, 1,2,3-triazole analogues of carbohydrate, Co/sulfonamide, Ag and Au/2-(2-thienyl)benzothiazole complexes exhibited an in vitro activity against resistant *Mtb* strains [147,148,149,150,151], but quickly degraded in cellular contexts [109,152,153]; furthermore, their entrapment in microemulsions [154] and nanomicelles [155] enhanced their bioavailability. Different types of Ga^3*^ complexes and rifampicin (Rf) in folate- or mannose-conjugated NPs were prepared in such a way as to enhance targeting of *Mtb*-infected macrophages [156].

More recently, the incorporation of Rf and the encapsulation into Ag NPs on the CS-g-(CA-MA-PZA) (amphiphilic chitosan-grafted-(acetyl alcohol-maleic anhydride-pyrazinamide) polymer has been carried out: these multi-drug delivery systems demonstrated the ability to enhance biocompatibility and cytotoxic effects on the cells [157]. More in general, the combination of polymeric NPs with antimicrobial inorganic systems is widely investigated: metal-based poly(lactic-co-glycolic acid) (PLGA) nanostructures were used to provide new properties to PLGA derivatives, such as antimicrobial, photo-thermal, magnetic, and bioimaging properties [158]. For example, the polyethylene glycol (PEG-PLGA/magnetite (γ-Fe_2_O_3_)) co-assembled with the antibiotic cotrimoxazole resulted in an excellent delivery system for the release of a drug via an external magnetic impulse [159]. Similarly, Au-conjugated PLGA-PEG-succinate-PEG-PLGA NPs assembled with Rf exhibited a time-controlled release useful for a long-term personalized therapy [160] and, similarly, Rf–Cu complexes were assembled in several structures with PEGs of different length [161].

Biodegradable multimetallic microparticles (MMPs) containing Ag and ZnO NPs were coated with Rf and successfully used for pulmonary delivery of anti-tubercular drugs to the endosomal system of *Mtb*-infected macrophages [162]. Furthermore, the combination of Rf with curcumin (CUR) in dual-loaded NPs demonstrated improved mycobacterial clearances [163]. On the other hand, multifunctional drug delivery systems Fe_3_O_4_/chitosan/isoniazid magnetic NPs with optimized size, excellent loading capacity, good magnetic properties, nontoxicity, and pH-triggered drug release property appeared applicable for tuberculosis treatment with excellent magnetic sensitivity and sustained release [164].

An innovative strategy is represented by polymeric dissolvable structures known as microneedles: they are able to create small holes in the stratum corneum to improve drug diffusion into the dermis joining the transdermal delivery ability of hypodermic needles with the advantages of dermal patches (safe and painless) [139,165]. Recently, hyaluronate (HA) microneedles have been investigated as possible vaccines against Tb; they allow for a time-dependent release of the Ag85B protein of *Mtb*, inducing a greater immune response in comparison with an intramuscular injection [166].

Similarly, self-assembly structures of peptides [167,168,169,170,171,172] improve stability and pharmacokinetic properties of AMPs and better internalize in host cells by endocytosis after conversion into vesicles [173]. Examples of this strategy are Linocin M18 and Lactacin F, which spontaneously form particles of 20–50 nm, or Iturin A, which is able to self-associate in vesicles of 150 nm in size [174]. On the other hand, there is the cationic peptide KSL capable of self-encapsulating into NPs retaining its antimicrobial activity for a long time against *Staphylococcus epidermidis* and *Staphylococcus aureus* bacteria [175].

## 4. Conclusions and Future Perspectives

The identification of new therapeutic strategies to counteract infections is the main challenge in medicine as recently pointed out by the wide spread of COVID-19 [178].

To address the drug resistance experienced in Tb infection, innovative strategies should explore the compounds alternative to current ones, but also combine new leads with non-standard delivery systems. Antimicrobial peptides can be considered valid candidates in the fight against *Mtb*, since they present unique features, especially for their highly specific MOAs that reflect into nano/micromolar K_D_ values toward their targets. But bacterial invasions are often very difficult to eradicate and the use of a high concentration of antibiotics has to be discouraged for their toxicity. The lack of newly discovered antibiotics prompt pharmaceutical research to enlarge the explored chemical diversity and metal-based antibiotics represent a wide and partially unknown world. In this review, we tried to outline the far-reaching impact of metal complexes on antibiotic drug discovery processes and also their potential use in combination with AMPs in *Mtb* infections. Metal-based compounds can be very versatile per se: their multiple MOAs due to different inertness of complexes and the tuning effects exerted by different coordination geometries both in redox and hydrophobicity features can pave the way to unexplored intervention ways. Anyway, to date, no metal-drug complexes have been approved by the FDA specifically for *Mtb* treatment, even if several transition metal compounds such as auranofin or ganite are FDA-approved drugs for other diseases and demonstrate a certain activity against *Mtb* [110]. In this context, the simultaneous optimization of cargo systems for the delivery of innovative antibiotics will lead to the identification of revolutionary treatments also increasing their market access.

## Figures and Tables

**Table 1:** Examples of recently developed (*2013–2020*) natural and chemically modified Antimicrobial peptides (AMPs) against Tb.

*Natural Peptides*				
AMP	Chemical Structure	Source	Activity	Mechanism of Action (MOA)
Pantocin wh-1 [22]	Cycle	*Pantoea dispersa* W18	Mycobacterium *smegmatis* mc^2^ 155, H37Ra mice	Unknown
Lassomycin [29]	Cycle	*Lentzea kentuckyensis*	*Mtb* and *Mycobacterium avium* subsp. *paratuberculosis*.	ATP-protease ClpC1P1P2
Bacteriocin AS-48 [30]	Cycle	*Enterococcus faecalis*	H37Rv, H37Ra, BCG Pasteur 1173, Mt103, CDC1551, GC 1237, H37Rv *phoP*, SS18b, *M. smegmatis* mc^2^ 155	Disruption of cell membranes
Micrococcin P1 [35]	Thio-cycle	*Staphylococci*	H37Rv	Inhibition of protein synthesis
Teixobactin [36]	Iso-cycle	*Eleftheria terrae* (β-proteobacterium)	H37Rv	Inhibition of cell wall synthesis
Ecumicin [37]	Cycle	*Nonomuraea* spp. MJM5123	H37Rv	Inhibition of ClpC1
Rufomycin I/Ilamycin A [38]	Cycle	*Streptomyces* sp. (MJM3502), *Streptomyces atratus* (NRRL B-16927)	*Mtb and Mycobacterium abscessus*	Inhibition of ClpC1
***Synthetic Peptides***				
Several cationic antimicrobial peptides (CAMPs) [23]	Cationic peptide rich in W and R	Peptide libraries	*M. smegmatis* mc^2^ 6, H37Rv and *Mycobacterium tuberculosis lux* strain	Pore formation
Cinnamic acid (Cin+) CAMP1, 2, 3, 5, 7 [24]	N-terminally modified protein	Five de novo proteins designed from CAMPs [23]	H37Rv, Multi drug resistant Tubercolosis (MDR-Tb)	Pore formation
hBD consensus hBD10 [25]	Disulfide bonds	Human β-defensin (hBD)	Multi drug resistant (MDR) strains, H37Rv	Possible activity on Kv channels
NZX [26]	Cysteine-rich protein	Plectasin	BCG, H37Rv	Unknown
*Vaejovis punctatus* Antimicrobial peptide (VpAmp1.0, 2.0) [27]	Cycle, disulfide bond	Mexican scorpion *Vaejovis punctatus*	MDR strains, H37Rv	Cell lysis
Cyclohexyl griselimycin (CGM) [28]	Cycle		H37Rv, *M. smegmatis* mc^2^ 155,	Inhibition of dnaN
Vesicle associated membrane proteins (VAMP) α1, α2, α4 (VapBC30) [39]	α-helix	Fragments of VapBC30	H37Rv	Inhibition of VapB30/VapC30
d-LAK 120 [40]	D-amino acid derivative, α-helix		MDR strains	Pore formation Inhibition of protein synthesis
LL37-analogous peptide(LLAP) [41]		LL-37	*M. smegmatis*	Inhibition of ATPase
Synthetic AMPs (SAMPs-Dma) [42]	Dimethylamination and imidazolation	De novo designed	*M. smegmatis* mc^2^ 155	Cell penetration DNA binding
Innate defense regulators ((IDR)-1002, -HH2, IDR-1018) [43,44,45]		Macrophage chemotactic protein-1 (MCP-1)	H37Rv, MDR strains	Immunomodulation Anti-inflammation
RNAse (RN3) (1–45) (RN6) (1–45) (RN7) (1–45) [46,47]		RNAse (RN)N-terminus	*Mycobacterium vaccae*; *Mycobacterium aurum*; *M. smegmatis* mc^2^ 155; *Mycobacterium bovis*; bacillus Calmette-Guérin (BCG)	Disruption of cell walls Cell agglutination Macrophage killing
Peptide B (Pep-B) [48]		hBD-1 (H β-Defensin-1)	H37Rv	Disruption of cell membranes Increase in host immunity
Synthetic cyclomarin A [49,50]	Cycle	Cyclomarin A	*M. smegmatis*, H37Rv	ClpC1 activity inhibition
Pandinin-2 (Pin2) based [51]	Short helix	Pandinin-2	H37Rv	Disruption of cell membranes

**Table 2:** Most common antimicrobial delivery systems.

Cargo System	Antimicrobial Compound	Bacteria
Liposomes	LL-37	*Escherichia coli* [141], *Pseudomonas aeruginosa* [176]
Ni NPs	LL-37	General Bacteria [142]
Au NPs	Surfactin, lactoferrin	*Staphylococcus aureus* and *E. coli* [98,144]
Ag NPs	Silver	*Mycobacterium bovis*, *Mtb* [146,177]
Micro and nano-emulsions	Ru complexes	*Mtb* [147,148,149,150,151]
Folate/mannose-conjugated NPs	Ga^3^* complexes, Rf	*Mtb* [156]
Polymeric NPs	CTZ, Rf	*Mtb* [159,160]
Dissolvable microneedles	Ag85B	*Mtb* [166]
Self-assembly nanoparticles	KSL peptide	*Staphylococcus epidermidis*, *aureus*
